# Usefulness of real-time PCR during follow-up of patients treated with Benznidazole for chronic Chagas disease: Experience in two referral centers in Barcelona

**DOI:** 10.1371/journal.pntd.0008067

**Published:** 2020-02-18

**Authors:** Elena Sulleiro, Aroa Silgado, Núria Serre-Delcor, Fernando Salvador, Maykon Tavares de Oliveira, Zaira Moure, Augusto Sao-Aviles, Inés Oliveira, Begoña Treviño, Lidia Goterris, Adrián Sánchez-Montalvá, Diana Pou, Israel Molina, Tomàs Pumarola

**Affiliations:** 1 Department of Microbiology, Vall d’Hebron University Hospital, Universitat Autònoma de Barcelona PROSICS, Barcelona, Spain; 2 Tropical Medicine Unit Vall d´Hebron-Drassanes, Universitat Autònoma de Barcelona PROSICS, Barcelona, Spain; 3 Department of Infectious Diseases, Universitat Autònoma de Barcelona, Vall d’Hebron University Hospital, PROSICS, Barcelona, Spain; 4 Departamento de Clínica médica, Unidade de Cardiologia, Faculdade de Medicina de Ribeirão Preto, Universidade de São Paulo, São Paulo, Brazil; Instituto de Ciências Biológicas, Universidade Federal de Minas Gerais, BRAZIL

## Abstract

**Background:**

Antitrypanosomal treatment with Benznidazole (BZ) or Nifurtimox may be recommended for patients with chronic Chagas disease (CD) to reduce the onset or progression of symptoms. However, such treatment has limited efficacy and high level of toxic effects. In addition, the current cure biomarker (serology conversion) precludes any treatment assessment unless a prolonged follow-up is arranged. PCR is thus the most useful, alternative surrogate marker for evaluating responses to treatment.

The aim of this study is to describe the usefulness of real-time PCR in monitoring BZ treatment within a large cohort of chronic CD cases in Barcelona.

**Methodology/Principal findings:**

A total of 370 chronic CD patients were monitored with real-time PCR post-BZ treatment. The median follow-up was 4 years (IQR 2.2–5.3y), with a median of 3 clinical visits (IQR 2–4). Only 8 patients (2.2%) presented with at least one incident of positive real-time PCR after treatment and were therefore considered as treatment failure. Four of those failure patients had completed full course treatment, whereas the remaining cases had defaulted with a statistical difference between both groups (p = 0.02). Half of the failure patients had undergone less than 4 years of follow-up monitoring all presented with parasitemia before treatment.

**Conclusions/Significance:**

BZ treatment failure was highly infrequent in our cohort. BZ discontinuation was a risk factor for positive real-time PCR results during clinical follow-up. Regular testing with real-time PCR during follow-up allows for early detection of treatment failure in patients with chronic CD.

## Author summary

Chagas disease has become a public health concern for health services in Spain, provided that the destination is the primary choice for Latin American migrants in Europe. Chronic phase is the most frequent clinical form of the disease outside of endemic areas. Most of these patients usually undergo treatment with Benznidazole, primarily childbearing women. Monitoring response to antiparasitic treatment without the presence of an early cure biomarker poses as one of the greatest challenges in managing this disease. In this study, almost 400 chronic Chagas disease patients treated with Benznidazole in two referral centers in Barcelona were included. Real-time PCR and serology were performed in all clinical visits after treatment; real-time PCR has been used as a surrogate biomarker to assess response to treatment. The failure rate of Benznidazole was very low in our cohort, with treatment discontinuation being a risk factor for such failure. In summary, our data supports that regular testing by real- time PCR for monitoring treatment response in chronic Chagas disease patients could be a useful surrogate marker for failure in such individuals.

## Introduction

Chagas disease (CD) is one of the most predominantly neglected diseases, particularly in Latin America where it is endemic [1]. It is estimated that 6–7 million people are infected by *Trypanosoma cruzi* globally, and between 68 000 and 120 000 of such individuals are living in Europe [2,3]. In Spain, CD has become a national health problem, as the country is the leading destination in Europe for Latin American migrants and the second in frequency worldwide [4].

The disease holds a varying clinical presentation, including a short acute phase characterized by high parasitemia and mild symptoms. Parasitological tests such as microscopic examination (including concentration methods like microhaematocrit and Strout test), blood culture, xenodiagnoses, or molecular techniques based on nucleic acid amplification (PCR) are the most commonly used techniques during this phase.

After acute phase, untreated patients experience the chronic phase with low and intermittent parasitemia. The clinical course is unpredictable, ranging from asymptomatic to severe disease with cardiac and/or gastrointestinal involvement. During such stage, diagnosis relies on serological techniques [5].

Specific treatment is always recommended for cases of acute CD, congenital transmission, reactivated infections, and children younger than 18 years [6]. Similarly, in spite of its varying efficacy and high level of toxic effects, treatment may be still indicated during the chronic phase to prevent the onset or progression of cardiopathy, as well as mother-to-child transmission [7–9].

For more than 40 years, only two nitroimidazole compounds, Benznidazole (BZ) and Nifurtimox, have been used as specific treatment for CD. BZ has considerable efficacy during the acute phase; however, cure rates during the chronic phase are lower and the number of treatment failures are incremental over time. In addition, BZ has an importantly broad spectrum of side effects, requiring the possible cessation of treatment [10]. In response to such fact, noteworthy efforts have been undertaken to develop new drugs. Yet, preliminary results based on randomized clinical trials, with PCR as a final endpoint, have discouraged their use and reinforced the effectiveness of BZ [11–13].

On a similar note, one of the main concerns concerning the follow-up of patients treated is the absence of an early cure biomarker. Serological clearance is the only cure biomarker as it stands; nevertheless, a decrease in antibodies is slow and requires an extensive follow-up [14]. Moreover, while the clearance of a parasitological test, including PCR, does not ensure the absence of infection, a positive result in treated patients does indicate treatment failure. As a result of its better accessibility in comparison to other parasitological tests, PCR is optimal for early detection of treatment failure and a reduction in follow-up time [15]. Detection of *T*. *cruzi* DNA by real-time PCR is currently the most useful surrogate marker for assessing response to treatment in both clinical practice and trials [16–17].

The aim of this study was to describe the usefulness of real- time PCR in monitoring BZ treatment within a large cohort of chronic CD patients outside of endemic area.

## Material and methods

This is a retrospective observational study performed in the Department of Microbiology at Vall d’Hebron University Hospital. The study population comprises patients with chronic CD visited between 2009 and 2016 in two referral centers: Vall d’Hebron University Hospital and the International Health Unit Vall d’Hebron-Drassanes. Patients were monitored every six months or annually with *T*.*cruzi* serology and real-time PCR. Follow-up data was collected until December 2018.

Inclusion criteria were: adults over the age of 18 with positive *T*. *cruzi* serology and treated with BZ. Real-time PCR was performed prior to the initiation of BZ treatment. Specific clinical contraindications including advanced cardiomyopathy, immunosuppressive condition, and pregnancy were considered as exclusion criteria.

BZ was administered orally in dosages of 5 mg/kg/day for 60 days, divided into three doses per day and with a maximum of 300 mg/day. All patients taking at least one dose of BZ were included in analysis.

Patients should have undergone at least 2 months of follow-up and provided at least one blood sample since the end of treatment.

DNA detection of *T*.*cruzi* by real-time PCR at any point after two months of BZ treatment was considered treatment failure.

### Microbiology procedures

For serological diagnosis, serum samples were analyzed in parallel for one recombinant antigen EIA (Bioelisa Chagas. Biokit, Lliçà d’Amunt, Spain from 2010 to 2015, and CHAGAS ELISA IgG+IgM. Vircell, Granada, Spain from 2015 to 2018) and one lysate antigen EIA (Ortho *T*.*cruzi* ELISA, Johnson & Johnson, High Wycombe, United Kingdom). For real-time PCR, blood samples were diluted at a 1:1 ratio with guanidine hydrochloride 6M and EDTA 0.2 M, pH 8.00 (Sigma Aldrich) at room temperature for 24 hours. DNA extraction was carried out with 200 μl through silica–membrane technology (NucliSens easyMAG. Biomerieux. France) and eluted in 50 μl according to manufacturer´s instructions.

A duplex real-time PCR targeted to *T*. *cruzi* SatDNA and human RNase P gene (Taq Man Human RNase P detection reagent; Applied Biosystems) was constantly performed in duplicate. From 2010 to 2013, real-time PCR reactions were carried out as described by Piron et al [18], using the TaqMan Universal PCR Master Mix. Amplifications were performed in a SmartCycler Real-Time PCR system (Cepheid, Sunnyvale, CA). Cycling conditions were a first step of 15 minutes at 95°C, followed by 40 cycles at 95°C for 15 seconds and 58°C for 1 minute.

From 2013 onwards, a protocol modification was introduced. The final concentrations in the real- time PCR mixture were 400 nM both primers and 100 nM TaqMan probe, using 5 μL of eluted DNA in 25 μL final volume. Polymerase was switched to Quantitec Multiplex PCR kit (Qiagen, Manchester, United Kingdom) and CFX Real–Time PCR detection system (Bio-Rad, Hercules, CA) was used for amplification.

In all cases, a sample was considered positive for *T*. *cruzi* when the threshold cycle (Ct) for the *T*. *cruzi* target was<40. If only one of the DNA targets amplified within > 40 and <45, a third amplification was carried out. It was considered positive when two out of three measurements were <40 Ct. A sample was considered valid when the internal control (RNase P human gene) was efficiently amplified (mean Ct 26 ±2).

Positive and negative samples were included in each real- time PCR run as external controls.

### Ethical clearance

Ethical approval for the study was obtained from the HUVH Ethics Committee. Procedures were performed in accordance with the ethical standards established by the Declaration of Helsinki as revised in 2013.

### Statistical analysis

Categorical data is presented as absolute numbers and proportions, and continuous variables are expressed as means and standard deviations (SD), or medians and interquartile ranges (IQR) depending on distribution. The Kolmogorov-Smirnov test was used to evaluate the normal distribution of variables. The χ2 test or Fisher’s exact test was used when deemed appropriate to compare the distribution of categorical variables; the t-Student test was employed to compare continuous variables. Results were considered statistically significant if the 2-tailed P value was <0.05. SPSS software for Windows (Version 19.0; SPSS Inc, Chicago, IL, USA) was chosen for statistical analyses.

## Results

A total of 370 chronic CD patients who had previously undergone BZ treatment were monitored with real-time PCR. Seventy percent of those patients (258/370) were women, and 95.4% (268/281) of them came from Bolivia. Forty-eight patients (48/280, 17%) presented gastrointestinal involvement, 21% (67/318) patients had a cardiac lesion, and 7% (19/272) present both gastrointestinal and cardiac involvements. Real- time PCR was performed before the initiation of specific treatment, testing positive in 39.7% (147/370) of all cases. The Ct values for the RNase P gene detection remained quite constant and had an acceptable signal.

The median follow-up time was 4 years (IQR 2.2–5.3y). The number of clinical attendance varied, with a median of 3 visits (IQR 2–4) per patient.

With respect to follow-up time, patients were divided into two groups: short term (<4 years) and long term (≥4 years). In the first group, 171 (46.2%) patients had a median of two visits (IQR 1–3), with a median follow-up time of 2.15 years (IQR 1.18–3.10 y). In the long-term follow-up group, 199 (53.8%) patients had a median of 4 visits (IQR 3–5), with a median follow-up time of 5.2 years (IQR 4.5–7.14 y).

BZ treatment was achieved in 84% (300/357) of the patients involved. Cutaneous symptoms accounted for the major cause of treatment discontinuation.

**Fig 1:**
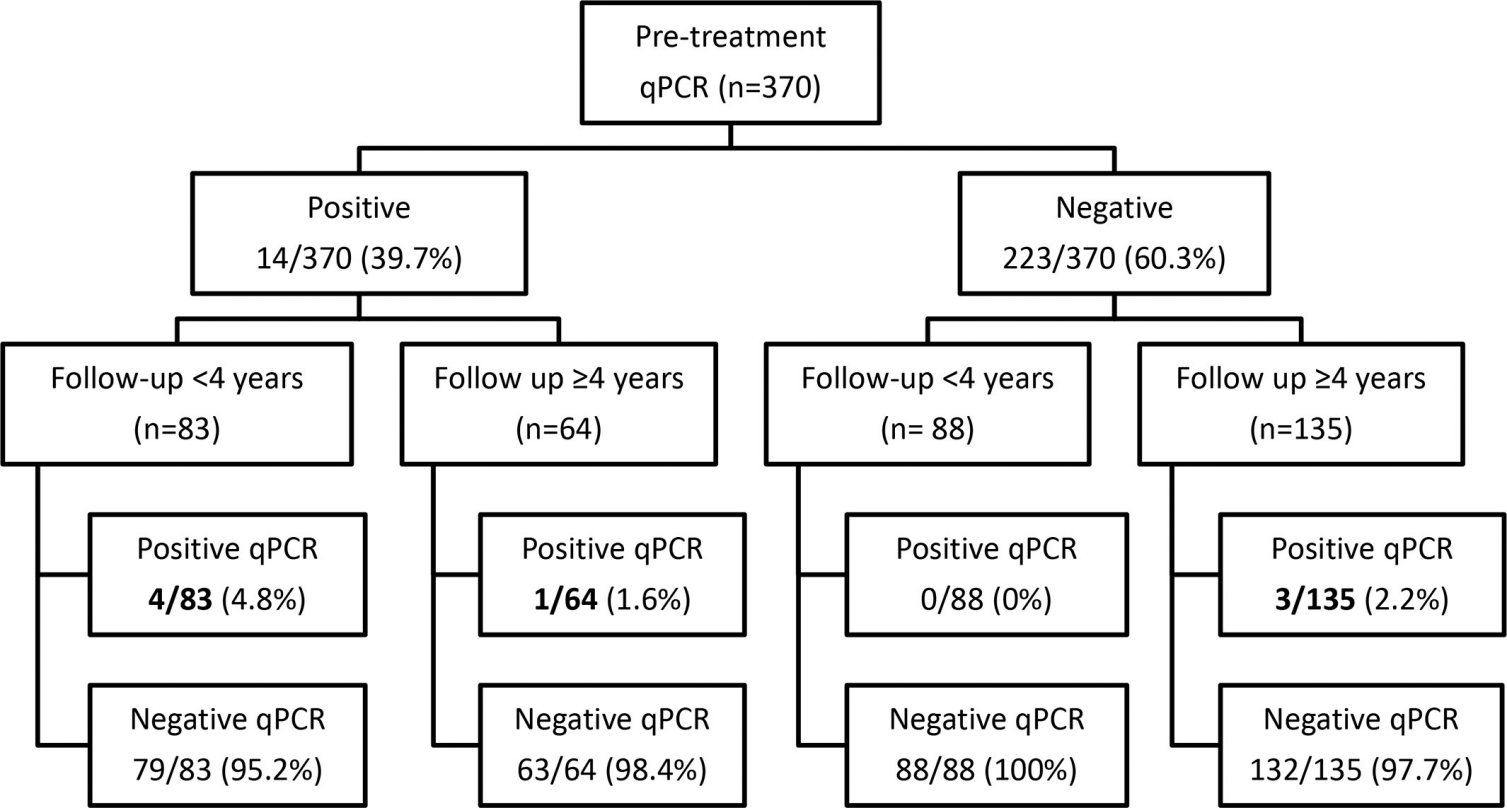
Flow diagram of the real- time PCR during follow-up.

During the study period, only 8 patients (2.2%) were considered as treatment failure after testing positive for least one *T*.*cruzi* real-time PCR. A flow diagram of the included patients is shown in Fig 1.

**Fig 2:**
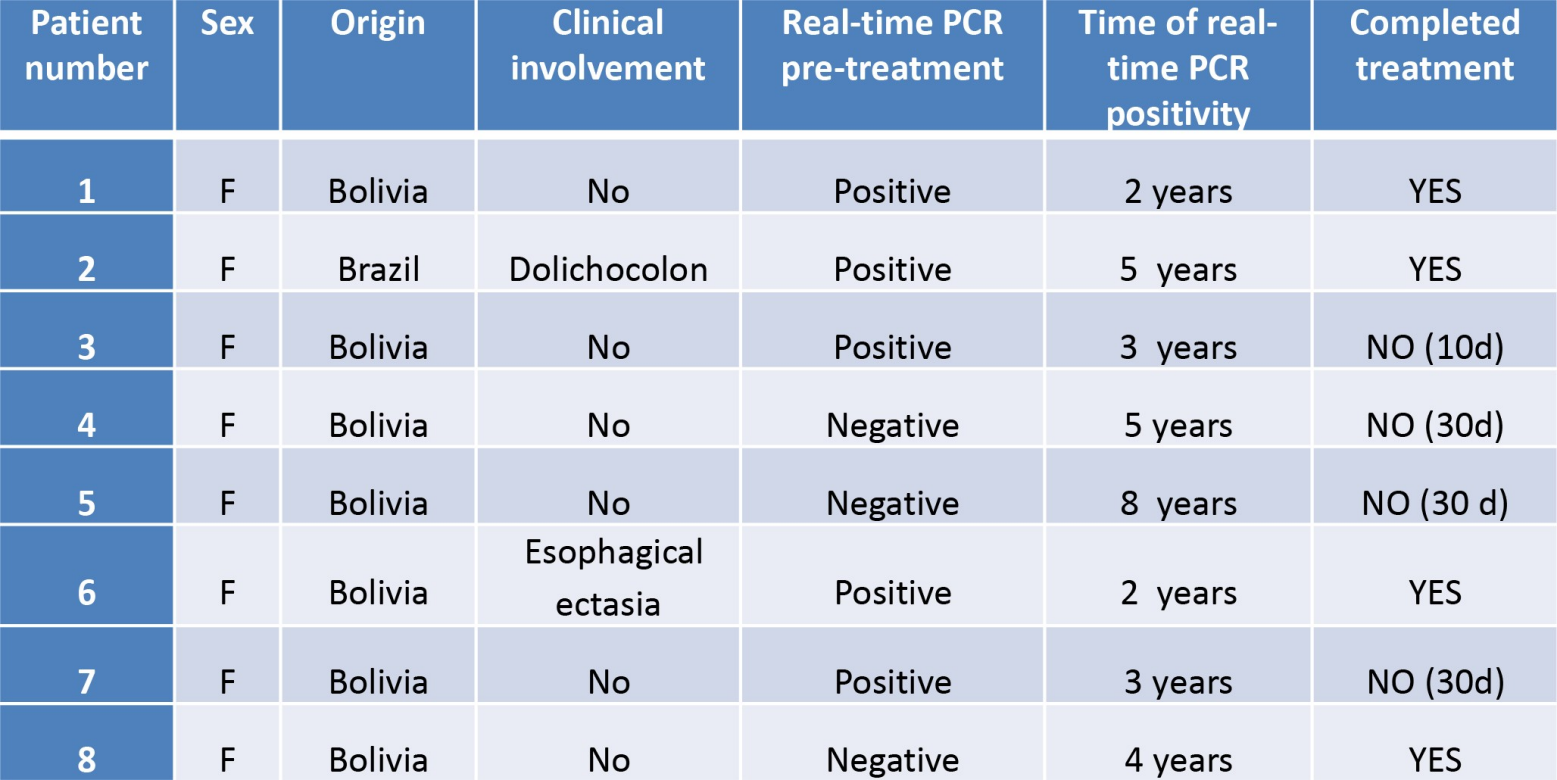
Clinical and epidemiological characteristics of patients with treatment failure.

Half of the failure patients had undergone a short-term follow-up (4/171, 2.3%) while the other half had been monitored during a longer period (4/199, 2%). Clinical and epidemiological characteristics of reported failure patients are summarized in Fig 2.

**Fig 3:**
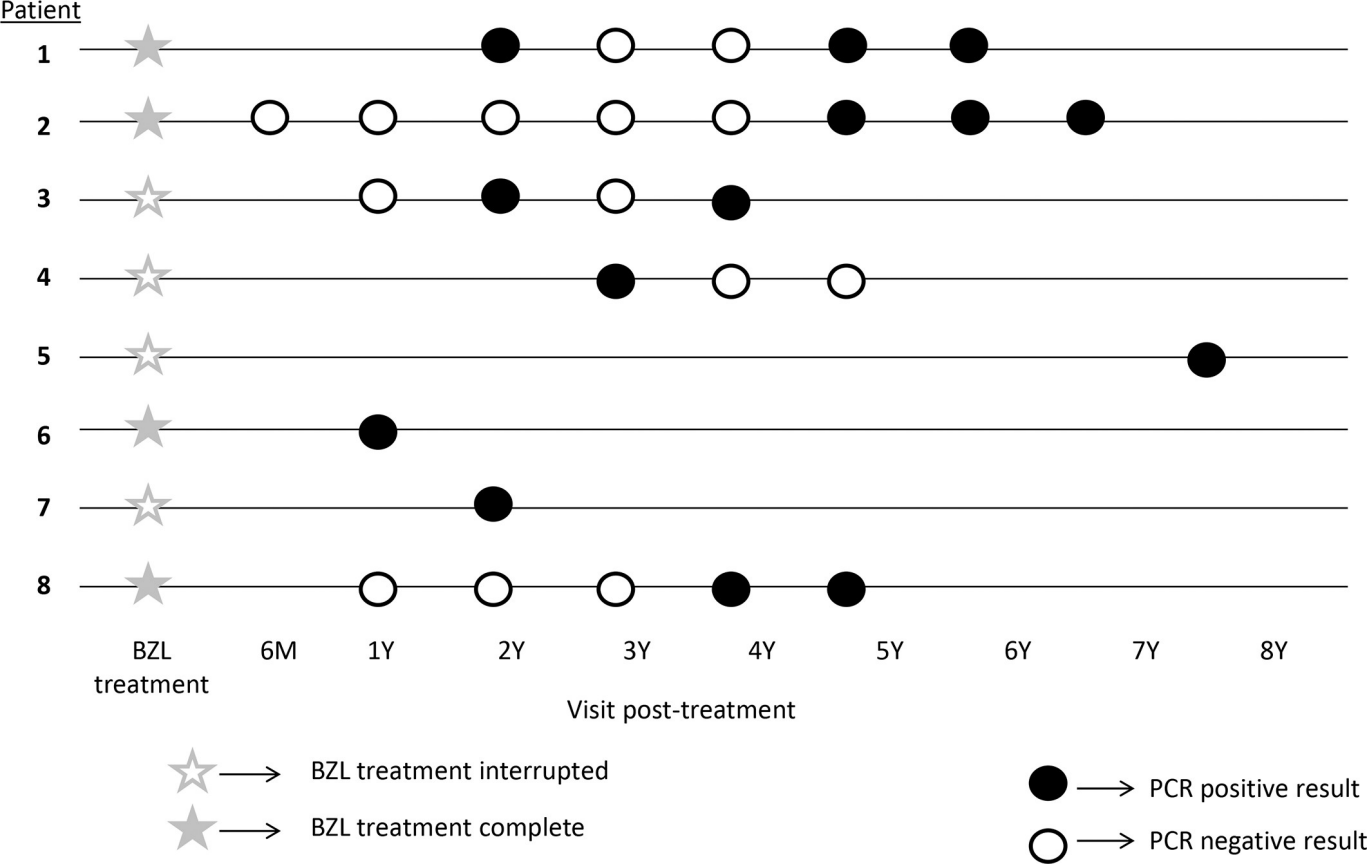
Chronogram of real- time PCR follow-ups in patients with treatment failure.

Five patients were followed after the detection of treatment failure. In three of them (cases 1, 3, and 4), post-treatment parasitemia was detected in several determinations intermittently. In the other two cases (cases 2 and 8), sustained real-time PCR positive result was recorded. The chronogram of real-time PCR follow-up in patients with treatment failure is represented in Fig 3.

Case 1 was included in the Chagasazol clinical trial (CHAGASAZOL ClinicalTrials.gov number, NCT01162967). Patient was randomized into the low-dose posaconazole arm (100 mg twice daily), with parasitemia being detected one year after treatment with posaconazole (11). A second treatment with BZ was administered. Unfortunately, two years after BZ treatment, parasitemia was detected once again. Patient is currently undergoing treatment with Nifurtimox.

Four failure patients (4/300, 1.3%) had completed correct treatment with BZ, while the remaining ones had an uncompleted treatment (4/57, 7%). Statistical difference was significant between both groups (p = 0.02).

Two hundred and seventy-five patients were monitored during follow-up with serology and real-time PCR simultaneously. Negative serology was only detected in 4 of those patients (4/275, 1.5%). No patient who tested positive for post-treatment parasitaemia showed negative serology during follow-up.

## Discussion

This study shows the results of 370 cases of treated chronic CD patients monitored with *T*. *cruzi* DNA detection by real-time PCR as a surrogate biomarker of treatment failure. Epidemiological and clinical features of our population were similar to other studies previously performed outside of the endemic area [19].

Real-time PCR was performed before the initiation of treatment in all patients, resulting positive in 40% of those individuals. Similar rates of positivity have been previously reported for untreated CD chronic patients outside of endemic areas [20–21]. Moreover, PCR sensitivity reported in several studies performed in Latin America achieved results higher than 60% in chronic patients [22]. The use of different PCR protocols; the likelihood of re-infection episodes; or parasitological factors such as heterogeneous distribution of different strains could explain this difference.

In our study case, only 2.1% of CD-treated patients had a positive real-time PCR result after BZ treatment. More than half of our patients had four-year follow-ups with regular clinical visits, strengthening our data. Similar results were observed in other study performed in Spain, where the positivity rate was 3.3% with a follow-up period of seven years [23].

Like pre-treatment PCR sensitivity data, our results show lower positivity rates after treatment in comparison to those of studies performed in endemic areas, with figures between 6% and 30% [24]. In two specific clinical trials carried out in Latin America, the level of failure reached was 46% in cardiopathy patients and 39.6% in children. In spite of this, the rate of failure results higher in the placebo group in both clinical trial [25–26].

It is known that the possibility for parasitemia detection after treatment increases over time. During treatment or even up to one year later, parasite clearance can reach to 100% [21]. However, after this early parasitemia decrease, an increase in real-time PCR could be detected over time [16]. Half of our patients had a positive real-time PCR result after four years, underscoring the importance of a long-term follow-up. An important difference was noted between patients treated with ergosterol inhibitors such as Posaconazole or E-1224, a ravuconazole prodrug. Some clinical trials performed with these promising drugs recorded failure rates around 90% for both posaconazole and E-1224 after a one-year follow-up [11–13]. Real-time PCR can thereby be also used to discard non-effective drugs in short-term follow-up.

Fifty-six percent of our patients had at least three clinical controls including real-time PCR in the follow-up. As real-time PCR is not a dynamic measure and only provides sampling information, serial monitoring increases the likelihood of detecting parasitemia. However, a sustained negative result of real-time PCR cannot discard failure over time.

Three failure patients presented with a negative real-time PCR result before treatment. This fact, could suggest that a pre-treatment negative real-time PCR result is not a good predictor of favorable response to treatment, mainly in areas with lower baseline PCR positive. However, in data reported by Torrico et al, patients with lower parasitic loads were more prone to present negative PCR after treatment [13].

These data are consistent with the low percentage of failures detected in our series, since all the patients presented high Ct. Nevertheless, this fact would not explain the cases of failure in patients with negative pre-treatment real-time PCR.

In our cohort, 16% of patients under BZ treatment were stopped due to adverse events. This result is similar to the 12.7% previously reported [27]. When considering the subgroup of patients who did not complete treatment, the rate of treatment failure increases from 2.2% to 7%: the difference is statistically significant with respect to the group of patients who did complete treatment. This data is in accordance with that reported in the cohort of Murcia et al [23]. In contrast, 20% of 81 Argentinean patients who discontinued BZ treatment showed a negative serology in a separate study [28].

Other factors could explain the treatment’s failure. The natural resistance of *T*. *cruzi* strains to the two drugs used to treat Chagas disease are known [29], as well as the pharmacokinetics of BZ in organic fluids, which may hinder a correct tissue biodistribution [30–31].

Some clinical trials are being performed using short dosage or duration of BZ therapy, or even intermittent treatment [32]. Optimizing BZ scheme in monotherapy or in combination with other antitrypanosomal drugs is one of the main goals in the current research surrounding chronic CD after the failure of ergosterol inhibitors.

In our cohort, only four patients (1%) achieved anti-*T*.*cruzi* antibody clearance during follow-up. Serological response monitoring can be useful to detect the proneness of antibodies to disappear. A good response to treatment could be considered when a sustained negative PCR result is detected and a decrease in antibody level is observed [23].

Clinical procedures and other treatment regimens after BZ failure are unclear; it poses as a challenge in the clinical management of these patients.

This study has some limitations. First of all, due to the retrospective nature of this study, clinical follow-ups were not regular. However, the use of real clinical practice data was an advantage in evaluating treatment efficiency. Real-time PCR protocol and serology test were changed during the study, but all controls and comparative studies reflected good correlation. Finally, the median time of follow-up is four years; a longer follow-up is needed to strengthen our findings.

## Conclusions

Currently, BZ is the most effective drug in treating chronic CD, with real-time PCR being the optimal surrogate marker for monitoring treatment failure over time. In our experience, regular testing with real-time PCR during follow-up allows clinicians to detect parasitemia both easily and early, and show treatment failure. Despite the four years of follow-ups, BZ treatment failure was infrequent in our cohort. Parasitemia was primarily detected years after treatment; BZ discontinuation is a risk factor of failure.

Important efforts should be undertaken in order to find both optimal CD treatment schemes with good effectiveness and fewer adverse effects, and better cure markers.
